# Discovery of an unrecognized pathway carrying overflow waters toward the Faroe Bank Channel

**DOI:** 10.1038/s41467-020-17426-8

**Published:** 2020-07-24

**Authors:** Léon Chafik, Hjálmar Hátún, Joakim Kjellsson, Karin Margretha H. Larsen, Thomas Rossby, Barbara Berx

**Affiliations:** 1 Department of Meteorology and Bolin Centre for Climate Research, Stockholm University, Stockholm, Sweden; 2 Faroe Marine Research Institute, Tórshavn, Faroe Islands; 3 GEOMAR Helmholtz Zentrum für Ozeanforschung Kiel, Kiel, Germany; 4 Christian-Albrechts-Universität, Kiel, Germany; 5 Graduate School of Oceanography, University of Rhode Island, Kingston, RI, USA; 6 Scottish Association for Marine Science, Aberdeen, Scotland, UK

**Keywords:** Ocean sciences, Physical oceanography

## Abstract

The dense overflow waters of the Nordic Seas are an integral link and important diagnostic for the stability of the Atlantic Meridional Overturning Circulation (AMOC). The pathways feeding the overflow remain, however, poorly resolved. Here we use multiple observational platforms and an eddy-resolving ocean model to identify an unrecognized deep flow toward the Faroe Bank Channel. We demonstrate that anticyclonic wind forcing in the Nordic Seas via its regulation of the basin circulation plays a key role in activating an unrecognized overflow path from the Norwegian slope – at which times the overflow is anomalously strong. We further establish that, regardless of upstream pathways, the overflows are mostly carried by a deep jet banked against the eastern slope of the Faroe-Shetland Channel, contrary to previous thinking. This deep flow is thus the primary conduit of overflow water feeding the lower branch of the AMOC via the Faroe Bank Channel.

## Abstract (web summary)

The authors show that overflow waters flowing toward the Faroe Bank Channel can take a previously unidentified path to the Faroe-Shetland Channel where it joins an unrecognized deep-reaching jet located along its eastern rather than its western boundary.

## Introduction

**Fig. 1:**
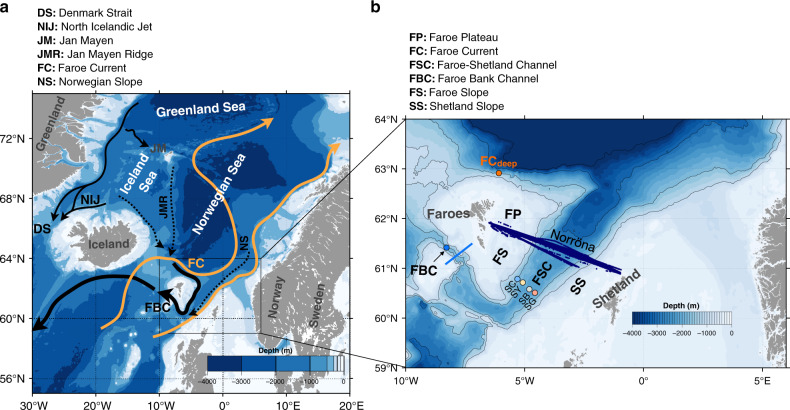
**Nordic Seas overturning circulation pathways**. **a** Bathymetric chart (shading; m) with the main upper-ocean (orange) and traditional deep (black) branches of the overturning circulation in the Nordic Seas. The dashed lines are deep branches suggested by earlier modeling studies to feed the Faroe Bank Channel^13,14^. **b** A zoomed map of the study region including locations of the multiple observational platforms used in this work. The circles refer to the deep Acoustic Doppler Current Profiler (ADCP) moorings (see Methods section). The light blue section is the hydrographic transect in the Faroe Bank Channel. The dark blue dots across the Faroe-Shetland Channel (FSC) indicate sites of velocity profiles from the vessel-mounted ADCP onboard MS Norröna. The black contours depict the 500, 800, 1500, and 2000 m isobaths.

The Atlantic meridional overturning circulation (AMOC) is a major regulator of the global climate system and its variability^1^. The AMOC conveys warm and saline Atlantic waters to higher latitudes, where they cool, sink, and return as dense overflow waters^2–4^. The Faroe Bank Channel Overflow (FBCO) (Fig. 1), which flows through a narrow passage across the Greenland–Scotland Ridge, is one of two key arteries (the other being the Denmark Strait, west of Iceland) of the AMOC transporting deep water from the Nordic Seas to the North Atlantic Ocean^5,6^. The volume transport of the FBCO has been monitored regularly since 1995 using bottom mounted ADCPs^7^ (see Methods section). These observations show that the FBCO represents about one-third (2 Sv) of the total overflow (5.8 Sv) across the Greenland–Scotland Ridge^8^. This extensive time series reveals that this deep branch feeding the AMOC is stable with no signs of any long-term slowdown^6,9^. Since the production of dense overflow water in the Nordic Seas is an important diagnostic for the stability of the AMOC^6,9,10^, any detectable change in the strength of the Nordic Seas overflows^11^ may be indicative of disruption of the main current systems in the North Atlantic and hence our climate.

Several studies have analyzed the wind and thermohaline processes influencing the time variability of the Nordic Seas overflow using both observations and models^7,12–18^. However, the source regions and the variable transport routes of the FBCO waters through the Nordic Seas have received limited attention^4,13,14,19^. In addition, most observational studies generally presume that the FBCO is fed solely by deep waters from the western Nordic Seas passing north of the Faroes and turning directly into the Faroe–Shetland Channel (FSC)^3,4,19^, Fig. 1. For example, the float study by Søiland et al.^19^ indicated that only water inside the 1750 m depth contour northwest of the Faroe plateau has the potential to be exclusively routed, under the Faroe Current^20^, to the Faroe Bank Channel via the FSC. However, the modeling study by Serra et al.^13^ suggested the existence of a two-branch system—one from the Norwegian slope and one along the Jan Mayen Ridge—that vary in strength depending on the wind forcing. This is somewhat in line with the modeling study by Köhl^14^ who, instead, wrote that the main source of the FBCO is the one along the Norwegian slope but with the tendency to become a two-branch system under strong cyclonic wind forcing with a smaller contribution from north of Iceland. To our knowledge, no dedicated observational study has yet demonstrated the existence of this eastern pathway from the Norwegian slope nor along which boundary in the FSC the bulk of the transport is routed before feeding the FBCO.

In this study, we investigate the existence, origin, and possible mechanisms that can activate this eastern overflow path using a combination of current measurements—moorings and vessel-mounted—and an eddy-resolving ocean model. In summary, our results reveal that overflow waters can also approach the FSC via an indirect path along the eastern margin, indicating that the western approach^19^ (short or direct path into the FSC) is not exclusive. Lagrangian analysis from the model further demonstrates that both pathways contribute to the FBCO, but the transport is enhanced when the eastern or indirect path is active and this depends on the prevailing atmospheric circulation via its regulation of the basin circulation in the Norwegian Sea. This study shows, for the first time, that the FBCO is, regardless of upstream pathways, primarily fed by a strong (and what seems to be a permanent) current jet at depth located, as seen in both observations the model, along the eastern rather than the western boundary of the FSC—hereafter referred to as the Faroe-Shetland Channel Jet (FSCJ).

## Results

### Inferring the eastern overflow path from observations

A strong indication that the flow feeding the FBCO does not always approach the FSC directly, as the study by Søiland et al.^19^ proposes, can be found in a deep current meter record moored underneath the Faroe Current at a bottom depth of 950 m^20^ (FC_deep_; see Methods section), Fig. 1b. Although this instrument is located at the upper edge (628 m) of the overflow-feeding dense water, the variability seen by the FC_deep_ is representative of bottom-intensified deep flows. To support the notion that the FC_deep_ is representative of the entire deep water transport under the Faroe Current, slope-ward of the 1750 m isobath, we utilize an ocean general circulation model (see Methods section). This model reproduces well the monthly to interannual variability of the FC_deep_ (Supplementary Fig. 1). The correlation between the observed and modeled eastward deep flow is consistently positive in the deep layers within the  ~2000 m isobath north of the Faroe slope (Supplementary Fig. 1), thus indicating that the FC_deep_ does not only capture a localized flow.

**Fig. 2:**
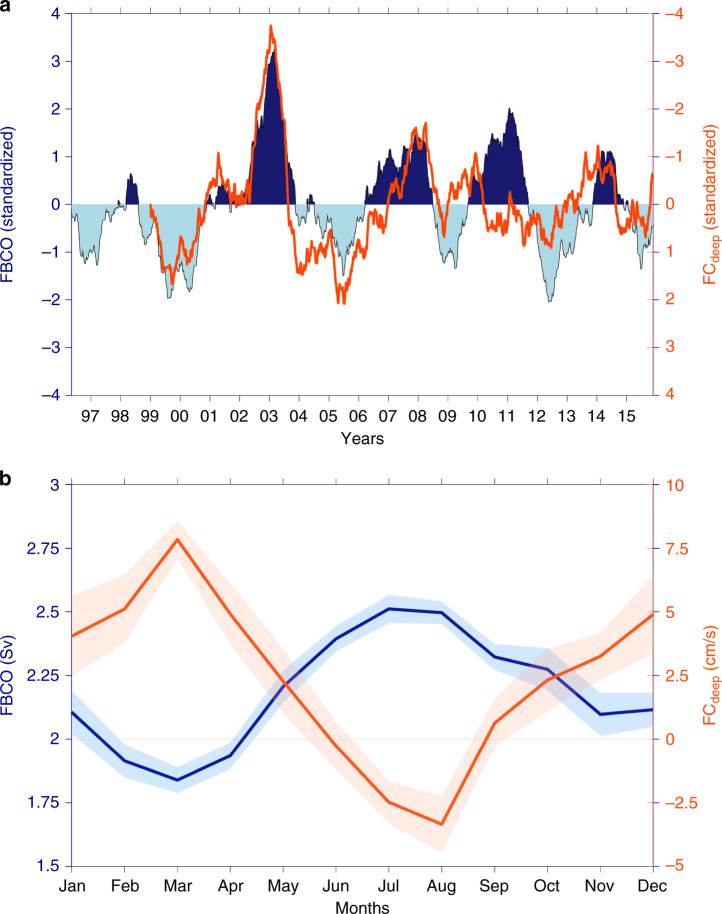
**Inferring the eastern overflow pathway**. **a** Standardized Faroe Bank Channel overflow (FBCO) for the 1996–2016 period (shading) overlaid by the standardized Faroe Current deep (FC_deep_, orange line). The axis of the standardized FC_deep_ is inverted. The time series have been detrended and smoothed with a 360-day running mean. The correlation coefficient between the time series is −0.69 (*p* < 0.01). The significance at the 99% confidence level is according to a random phase test^35^ using 20000 Monte Carlo simulations. **b** Seasonal cycle of the FBCO (blue; Sv) and FC_deep_ (red; cm s^−1^). Positive transport of the FBCO means southwards into the North Atlantic Ocean. Positive/negative velocities of the FC_deep_ mean eastwards/westwards. The shading denotes the standard error of the mean.

The results show a strong anticorrelation between the FC_deep_ and the FBCO transport time series on both interannual (Fig. 2a) and seasonal time scales (Fig. 2b) for the 1999–2016 period (note, however, that the relationship on interannual time scales is weaker since 2008, see discussion in ref. ^18^). This inverse relationship, i.e., a strong FBCO transport coinciding with a weak or even reversed FC_deep_ (Supplementary Fig. 2), suggests that the FBCO cannot exclusively originate from the traditionally assumed western approach^19^ or direct path into the FSC. Instead, it points to a second or alternate pathway by which the FBCO is fed. It is, however, important to note that the general circulation in the Norwegian Sea is clearly cyclonic^21^ such that in both cases the source waters produced in the western Nordic Seas must pass north of the Faroes; the eastern Nordic Seas cannot be a source for the dense overflow waters as suggested by ref. ^14^. It is equally important to note that the study by Søiland et al.^19^ was a single realization and the intermittent eastern path may have been missed as a result. In conclusion, these observational results help to infer that an alternative path, via a longer loop from north of the Faroes to the eastern boundary in the Norwegian Sea, as the above-mentioned model studies^13,14^ hint at, must also be feeding the FBCO and hence the lower branch of the AMOC.

### Mechanisms activating the eastern overflow path

**Fig. 3:**
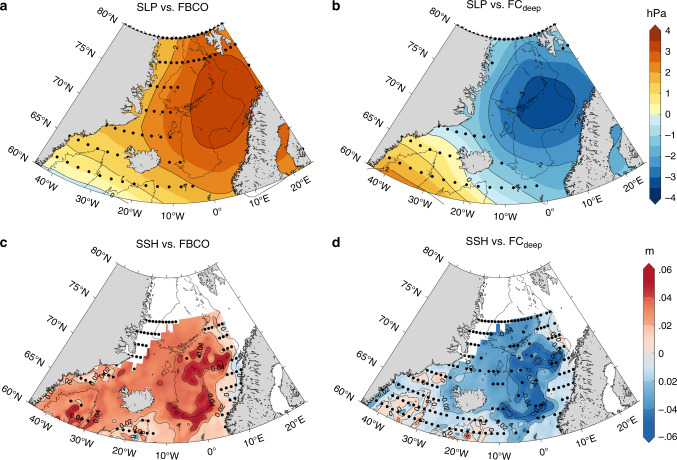
**Large-scale fingerprints of the deep flows**. Composite difference analysis of sea-level pressure (SLP; **a**, **b**) and sea-surface heights (SSH; **c**, **d**) derived from satellite altimetry. The analysis is based on the difference between periods when the Faroe Bank Channel overflow (FBCO, left) and Faroe Current deep FC_deep_, right) time series are larger and smaller than one standard deviation. The data have been detrended and smoothed with a 360-day running mean before the analysis. The spatial patterns point to the close anti-phase relationship between the FBCO and FC_deep_. The SLP spatial patterns show that anticyclonic atmospheric circulation anomalies over the Nordic Seas lead to stronger FBCO and weaker eastward directed FC_deep_, and vice versa. The SSH spatial patterns highlight that the importance of the wind-driven barotropic flow along closed *f*/*H* (*f* is the Coriolis parameter and *H* is depth) contours in the Nordic Seas. The gray line depicts the 2000 m isobath. White is missing data.

Since the western approach cannot be the sole source for the FBCO, we must ask what could potentially drive this alternative path. We note that when the atmospheric circulation governing the Nordic Seas is, on interannual time scales, anomalously cyclonic (anticyclonic), the FBCO transport weakens (strengthens), Fig. 3a. At the same time, the FC_deep_ is found to strengthen (weaken) indicating that this alternative pathway is associated with an anomalously anticyclonic atmospheric circulation regime (Fig. 3b) via its regulation of the wind-driven basin-scale circulation in the Norwegian Sea as seen in sea-surface heights derived from satellite altimetry (Fig. 3c, d). Even in the seasonal cycle (Fig. 2b), weakened (strengthened) cyclonic winds during summer (winter) can also explain the high (low) FBCO transport and the low or reversed (high) velocities of the FC_deep_, suggesting a fast barotropic response to wind forcing (see also ref. ^18^).

**Fig. 4:**
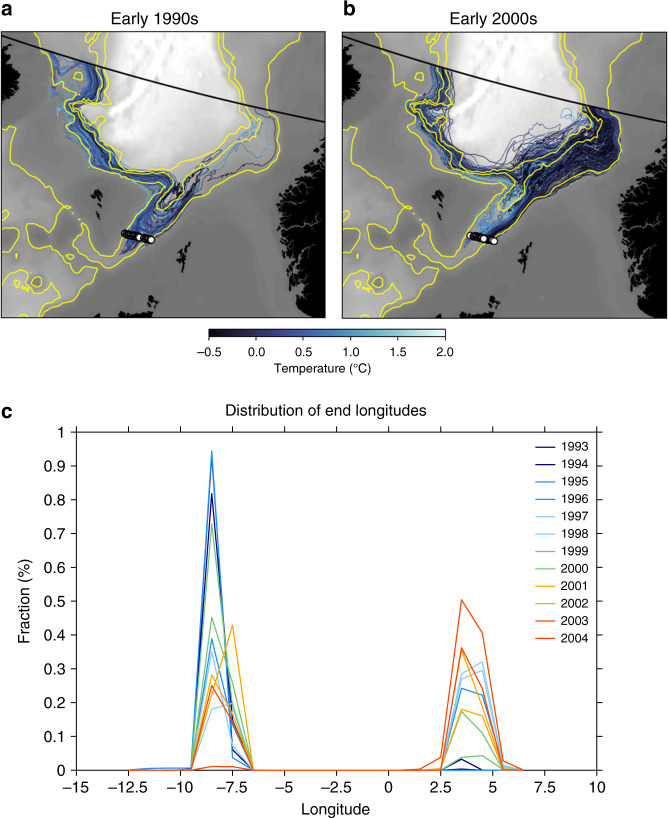
**Capturing the two main pathways feeding the overflow**. **a** Backward trajectories released in the Faroe-Shetland Channel (FSC, white dots) in **a** 1994, and **c** 2003 and backtraced for five years. The colors in both **a** and **b** indicate that only waters colder than 2 °C are traced. The yellow contours depict the 800, 1600, and 2000 m isobaths. **c** Probability density function of the fraction reaching the northern boundary (black line in **c**/**d**) after one year. The blue lines represent the early 1990s period, while the red lines represent those of the early 2000s. Note, for example, during the early 1990s, most of the particles exit the domain within one year in the western part of the boundary. While during the early 2000s, particles are also seen to approach the FSC from the Norwegian slope along the eastern boundary.

To investigate more closely the pathways excited by the different atmospheric forcing, we employ backward trajectory simulations (see Methods section) to identify the variable modeled pathways feeding the FBCO. To ensure that we are only tracing the densest overflow water, we only track water particles colder than 2 °C and only do so until they reach a latitudinal section corresponding to  ~ 65°N (Fig. 4). Backtracking water for several years is important, not only to demonstrate the robustness of the variable pathways, but also to show the significant role a multiyear atmospheric forcing regime plays in modulating the modeled FBCO pathways. In this regard, it is instructive to trace these deep dense waters during the early 1990s and 2000s (Supplementary Fig. 3), as these are periods with particularly strong and weak wind forcing and hence spin-up and spin-down of the top-to-bottom basin circulation in the Norwegian Sea^21^ (Fig. 3), respectively. Figure 4a demonstrates that when the atmospheric forcing, and hence the basin circulation in the Norwegian Sea is anomalously cyclonic (early 1990s), the FBCO is weaker than normal and the source of the deep water is predominantly via the western approach, the short or direct path around the Faroe Plateau into the FSC. In contrast, when the atmospheric circulation is in an anticyclonic regime (early 2000s), the FBCO is stronger than average and the path is predominantly along the eastern Norwegian Basin (Fig. 4b), the long or indirect path into the FSC. Thus, fluid is deflected from north of the Faroes over to the Norwegian slope before turning south into the FSC. There is, however, a subtle difference between the two periods: while the trajectories during the early 1990s seem to be more constrained to shallower depths, those pertaining to the early 2000s appear to trace deeper isobaths and hence a second path is opened up with water crossing from north of the Faroes over to the Norwegian slope.

The two distinct modeled pathways of deep water are further quantified by the distributions of final longitudes for the backward trajectories (Fig. 4c), which are clearly bimodal but also exhibit distinct year-to-year variability in magnitude. Strengthened cyclonicity during the early 1990s (anticyclonicity during the early 2000s) of the atmospheric circulation within the Nordic Seas and therefore that of the Norwegian Sea produces an unimodal (bimodal) probability distribution suggesting that under anomalously cyclonic (anticyclonic) wind forcing the direct (indirect) path contribution to the FBCO is dominant. These model results strongly indicate that the prevailing atmospheric circulation via its regulation of the wind-driven basin circulation is able to draw upon different upstream deep water pathways feeding the FSCJ (as discussed below) and ultimately the FBCO: one is routed directly into the FSC, and one reaching the Norwegian margin before turning south toward the FSC. Note, however, that the direct path does not disappear entirely when the indirect path develops: the FBCO sees the combined flow from both branches but there is a preference and not a simple case of either-or. It is equally important to note that the Lagrangian analysis only demonstrates two pathways (Fig. 4c), which is consistent with that water must come from depths comparable to the Faroe Bank Channel sill (~840 m). This dynamical requirement thus renders only two options: The western slope (direct path) and the eastern slope (indirect path).

### Is the eastern boundary the major route for the overflow?

**Fig. 5:**
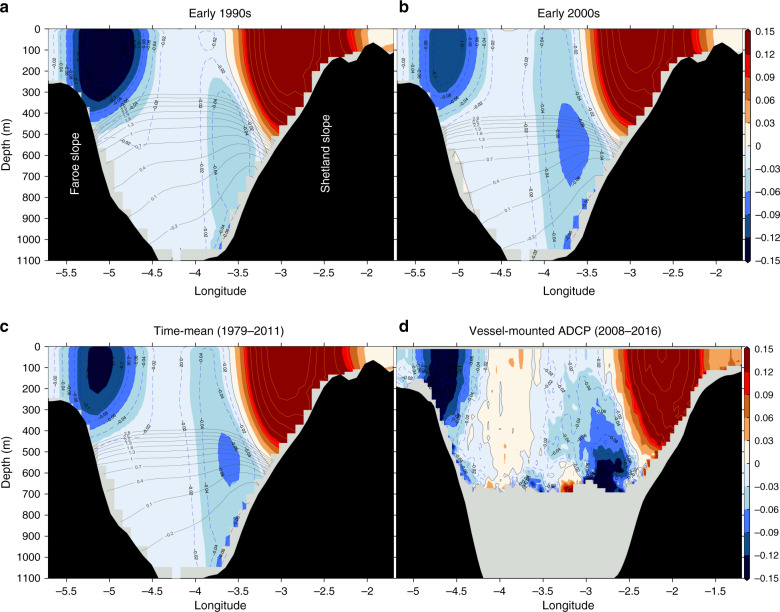
**Capturing the deep eastern jet in the FSC**. Modeled velocities (southwards/northwards correspond to red/blue shading) across the Faroe–Shetland Channel (FSC) (Supplementary Fig. 1, inset) averaged for **a** the early 1990s, **b** the early 2000s, and **c** 1979–2011 period (model climatology). The gray lines show the corresponding isotherms but only less than 2.8 °C (black shading is bottom topography). **d** The observed 2008–2016 velocities from the vessel-mounted Acoustic Doppler Current Profiler (ADCP, cf. Fig. 1b). Missing data below 600 m is indicated by light gray. The Faroe–Shetland Channel jet (FSCJ) is banked against the eastern slope as indicated by the sloping isotherms (thin gray lines). It is also strongly bottom-intensified and has another a core at intermediate depths. The overall structure of the FSCJ is similar between the modeled mean velocity and that based on the 9-year long ADCP data (2008–2016) from Norröna (see Methods section).

**Fig. 6:**
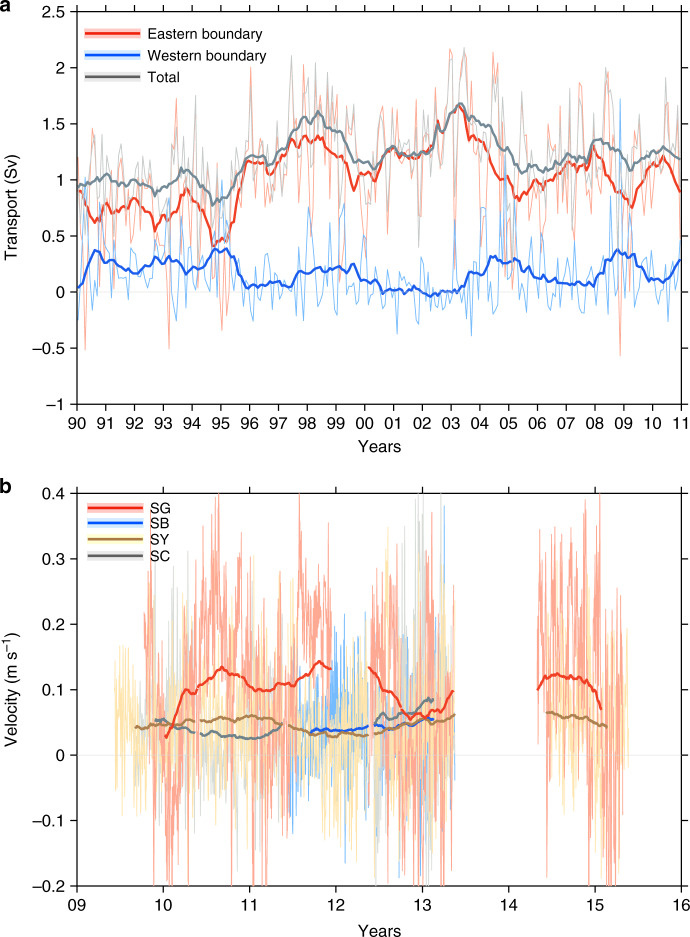
**The deep jet is the main conduit of the overflow**. **a** Volume transport (positive means southwards into the North Atlantic Ocean) estimated from the model for the western and eastern boundaries in the Faroe–Shetland Channel (FSC) (Supplementary Fig. 1, inset) and for depths deeper than  ~500 m (see Methods section). **b** Velocities (positive means southwards) from the Acoustic Doppler Current Profiler (ADCP) moorings stretching across the FSC on both daily (thin) and interannual (thick, 360-day running mean) time scales. Figure 1b shows the location of the ADCP mooring profiles sites in the FSC. Note that the Faroe–Shetland Channel jet (FSCJ) is strongest in both the model and the SG mooring (red line).

A close look at the meridional velocities in the FSC (Fig. 5) suggest, however, that even when the direct path dominates, the main source feeding the FBCO is along the eastern boundary of the channel (Shetland slope), and not along the Faroe slope as has long been assumed^4^. Figure 5 shows that the strongest modeled deep velocities, and hence the bulk of the FBCO transport (Fig. 6 and Supplementary Fig. 4), are found at the eastern boundary for both the early 1990s (Fig. 5a) and 2000s (Fig. 5b) and therefore also for the mean state (Fig. 5c). This mean structure, with a bottom-intensified FSCJ, is reinforced by the 9-year long record of along-channel velocities obtained from ship-mounted ADCP^22^, Fig. 5d, suggesting that this is indeed a robust overflow pathway through the FSC. This observed mean state supports the notion that overflow waters, even when originating from the west, tend to cross over to the eastern boundary after entering the channel (It is possible that the shape of the bathymetry, cf. Fig. 1b, plays a role in guiding the flow to the eastern boundary), and indeed clear evidence of this behavior can be seen in the float trajectories reported in refs. ^19,23^.

To further confirm that the FSCJ carries the bulk of the FBCO, we calculate the modeled volume flux of the deep waters (see Methods section) in both the western and eastern boundary currents of the FSC (Fig. 6a). The results demonstrate that the FSCJ, in a time-mean sense, accounts for the major volume transport through this channel, while at the western boundary the transport is small. The time variability of the modeled volume transport associated with the FSCJ further suggests that the early 2000s was indeed a period of record high overflow, and that the transport during the early 1990s were comparatively weaker; a finding consistent with the observed record high FBCO and record low FC_deep_ during the early 2000s (Fig. 2a). Furthermore, ADCP moorings across the FSC (see Methods section) confirm, since they reach deeper than the ship-mounted ADCP (Fig. 5d), that the highest deep southward velocities are found in the eastern rather than the western boundary or any other mooring location in the FSC (Fig. 6b). We conclude, based on both model simulations and observations from multiple platforms, that the FSCJ is the main conduit of overflow waters through the Faroe Bank Channel regardless of upstream pathways.

## Discussion

A key difference between the two periods under investigation is that according to the model during the early 1990s, the flow is strongly constrained to shallower isobaths that connect directly to the FSC, while during the early 2000s an additional flow to the east opens up along deeper isobaths (Fig. 4b). This water, after passing north of the Faroes over to the Norwegian slope, turns south and continues into the FSC. It is very likely that this deeper route reflects an increased supply of water originating from along the Jan Mayen Ridge (Fig. 4a), while during the 1990s water come predominantly from north of Iceland along a shallower route or shallower isobaths connecting directly to the FSC (Fig. 4a). Thus, the different wind-forcing conditions in the Nordic Seas causes water to be drawn from preferentially different pathways (Jan Mayen Ridge vs. north of Iceland) and reach the FSC along different depths or routes (deeper vs. shallower); a result that also provides an insight into the inverse relationship between the FCdeep and the FBCO. It is also worth-stressing the fact that the large-scale patterns (Fig. 3) associated with the FBCO transport and FC_deep_ bear a strong similarity further supports the notion that the FC_deep_ is not a local feature but is more representative of the basin-scale circulation in the Norwegian Sea.

**Fig. 7:**
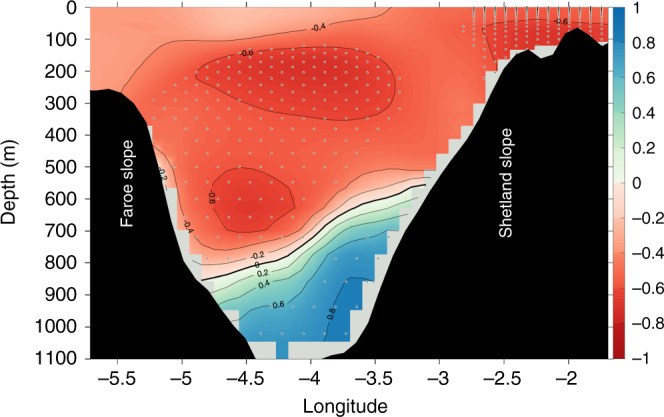
**Enhanced deep jet coincide with increased overflow density**. Longitude-depth correlation pattern between annual mean Faroe–Shetland Channel jet (FSCJ) volume transport and density variations across the Faroe–Shetland Channel (FSC) (Supplementary Fig. 1, inset) for the 1990–2010 period. Positive correlation means that a stronger FSCJ coincide with higher overflow density. This correlation pattern outlines the structure of the FSCJ, with the maximum correlation located at depth in the eastern boundary of the FSC. Stipplings indicate correlations significant at the 95% confidence level according to a random phase test^35^ using 20000 Monte Carlo simulations.

So far little has been said on the consequences such a two-branch overflow system would have on water mass properties, although the Lagrangian particles that reach the FSC via the indirect path from the Norwegian slope are seen to be much colder than those entering the channel via the direct path (cf. Fig. 4a, b). This is evidently the case for the early 2000s, a period when the eastern pathway is activated and the FSCJ transport is anomalously strong. Correlation analysis between the modeled density variations across the FSC and the FSCJ transport variations further supports this view, Fig. 7. The latter shows that the modeled FSCJ transport is strongly positively correlated with deep water density, which reinforces that the indirect path not only coincides with stronger FSCJ transport but also leads to denser waters feeding the overflow through the Faroe Bank Channel. This unanticipated bimodality of deep water pathways through the southern Norwegian Sea thus has implications for the Iceland–Scotland overflow water and hence the state of the subpolar overturning circulation^10^.

Although suggestive evidence for deep southwestward flow along the Shetland slope can be found in several previous studies^16,24^, the existence of the FSCJ has not been demonstrated before ref. ^16^ found, using an ocean general circulation model, an antiphase relationship between the FBCO transport and the slope current in the Norwegian Sea, but without noting the presence of a southward flow connecting the two; perhaps it went unrecognized because the FSCJ flows at depth along the outer edge of the slope current (Fig. 5). Our discovery that the FSCJ—a deep-reaching current that flows along the eastern boundary in the FSC—carries the bulk of the FBCO transport completely alters our view of the flow dynamics in the FSC. It does so in a similar way that the North Icelandic Jet^25,26^ (cf. Fig. 1a), which we recognize bears strong resemblance in its structure and location to the FSCJ, has done for the Denmark Strait overflow. Although we have established that, regardless of upstream pathways, the bulk of the FBCO will be carried by the FSCJ, further work beyond the scope of this study is required to understand the involved dynamics and sensitivity to changing forcing in future climate.

In summary, we have in this study showed evidence of an eastern path feeding the overflow through the Faroe Bank Channel from both observations and an eddy-resolving ocean model. The deep current north of the Faroes, i.e., FC_deep_, underneath the main core of the Faroe Current is found to be closely connected to the FBCO and key in inferring the eastern overflow path from observations. The FC_deep_ showed a weakening in the early 2000s (even a reversal during 2002–2003), while the FBCO was concurrently experiencing the highest transports on record. This observed antiphase relationship, which holds on seasonal as well as on interannual timescales, could not be reconciled, pointing to an alternative pathway feeding the FBCO along the eastern slope of the southern Norwegian Sea. This perspective was validated though a Lagrangian approach using an eddy-resolving ocean model forced by realistic winds. The Lagrangian analysis further suggested that the prevailing atmospheric regime via its regulation of the basin-scale circulation is key, not only in forcing anomalous FBCO years as previously discussed^13,18^, but also in dynamically activating a previously unrecognized overflow path along the eastern Norwegian Sea. Finally, based on the modeled transport, ship-mounted and moored ADCPs, we have established that the water feeding the FBCO is routed through the FSC along its eastern rather than western boundary, regardless of upstream pathways in the Nordic Seas. The FSCJ is thus the main conduit of overflow water through the Faroe Bank Channel and hence connects to the lower branch of the AMOC.

## Methods

### Observations of Faroe Bank Channel overflow

Since November 1995, the FBCO has been monitored continuously (except for annual 2–3 week servicing breaks) by an upward-looking 75 kHz RDI BroadBand ADCP moored close to the bottom centrally in the Faroe Bank Channel. This has been complemented by additional shorter term mooring deployments at each side of the sill, and regular conductivity-temperature-depth cruises, conducted 3–4 times each year. The time series used in this study, i.e., the kinematic overflow, is based on the data from the long-term central ADCP, which by analysis of the complete data set has been shown to be representative of the volume transport of the deep flow and approximately proportional to the volume transport of FBCO defined in other ways^7,9^ (e.g., *σ*_θ_ > 27.8 kg m^−3^).

### Measurement of the deep flow north of the Faroes

As part of a long-term monitoring effort of the inflow of warm Atlantic water to the Nordic Seas, an upward-looking 75 kHz RDI BroadBand ADCP has been moored at a location (62.92°N, 6.08°W) north of the Faroes with bottom depth around 950 m^20^. From the ADCP measurements, a time series of daily averaged horizontal velocity was generated by interpolation. The time series represents velocities at a depth of 628 m, which is located beneath the Atlantic water layer associated with the Faroe Current. The time series commenced in July 1998 and is continuous except for the annual 2–3 week servicing breaks.

### Current velocities from the ADCP mounted on MS Norröna

Velocity transects across the FSC (cf. ref. ^27^, their Fig. 2b) are obtained by means of a 75 kHz RDI Ocean Surveyor ADCP mounted in the hull of the high seas ferry MS Norröna. The ADCP operates in the narrow-band mode to reach as deep as possible,  ~600 m, although the data returns drop rather sharply beyond  ~500 m depth. For our analysis the single ping profiles are averaged every 3 min to provide along track sampling of currents every 3 km (at a vessel speed of 20 Kt) with a vertical resolution of 16 m and an uncertainty of  ±0.02 ms^−1^. A Thales ADU-5 with 10 m antenna separation (8 m fore-aft and 14 m port-starboard) provides vessel heading once per second with an accuracy 0.03^∘^T, which at 20 Kt translates into a cross-track error of  ~0.005 ms^−1^. All velocity data between 2008 and 2016 have been detided prior to analysis. Additional information can be found in refs. ^22,28^.

### Current velocities from moored ADCPs in the FSC

The FSC Transport Mooring Array, a collaboration between Scottish, Faroese, Norwegian, and German scientists, collects moored current velocities at several locations across the FSC. Here, data from the S-line of moorings^29^ (Fig. 1b) has been used to investigate the circulation deeper than 600 m. The mooring SG/SC/SY/SB is located at a bottom depth of 1044/1069/909/786 m and the depth of its deepest bin is at 810/617/668/642 m. More information on data collection can be found in ref. ^29^.

### Atmospheric reanalysis

We use both the daily and monthly mean sea level pressure data from ERA-interim^30^ provided by the European Center for Medium-range Weather Forecasts. The fields have a horizontal grid resolution of 1° × 1° and span the 1993–2016 period.

### Ocean model and trajectory code

We use the three-dimensional velocity, temperature and salinity fields from a simulation of the global ocean using the NEMO ocean model^31^, version 3.6. The simulation, ORCA0083-N001, uses a global grid of nominally 1/12° horizontal resolution and 75 vertical z-star levels. We choose to use a relatively high-resolution model since it is able to explicitly resolve much of the mesoscale eddy–eddy and ocean–atmosphere interactions, unlike lower resolution models, where such processes are not well represented^32^. The ocean model is forced by the DRAKKAR forcing set v5.2, which is based on the ERA-40 and ERA-interim reanalysis for the 1958–1978 and 1979–2010 period, respectively. The simulation is free-running except for a restoring of sea-surface salinity toward climatology. The model was started from rest in 1958 and run until 2010, but the model output before 1979 is discarded as a model spin-up.

We use the TRACMASS Lagrangian trajectory code v6.0^33^ to trace water masses backwards in time from the FSC. Numerical particles are seeded every five days in all grid cells of the section channel, where the overflow is transported, and are then traced backward in time until they either cross the northern boundary of our domain  ~65°N or until they leave the domain (Fig. 4a, b) or until five years have passed. For our statistics (Fig. 4c), we exclude trajectories that remain south of the northern boundary ~65°N after one year (note, however, that the results are not sensitive to the arrival times of the particles, Supplementary Fig. 5) and also trajectories that reach a temperature above +2 °C. These criteria allow us to clearly isolate the trajectories representing deep water in our analysis.

### Satellite altimetry

We utilize daily multimission satellite altimetry^34^ (DUACS DT2014) to study the sea-surface height spatial patterns associated with the FBCO transport and the FC_deep_ (Fig. 3). The grid resolution is 0.25° × 0.25° and the period under investigation is between January 1993 and April 2016.

### Volume flux

The model volume transport calculation across the FSC is defined as follows:

1 $$\varPsi (t)=\int_{0}^{L}\int_{-h(x)}^{482}v(x,z,t){\mathrm{d}}z{\mathrm{d}}x,$$

where *v* is the along-channel velocity component, *L* is width of section, *x* and *z* are the along-section distance and the depth, respectively, *h*(*x*) refers to bottom depth, and *t* is time. This flux calculation is done for both the western and eastern parts of the channel, which here are separated by the deepest part of the channel. The flux calculation has also been done for distinct layers below 482 m to demonstrate, where the largest transport is found (Supplementary Fig. 4). The model transport calculations were done with the CDFTOOLS package (https://github.com/meom-group/CDFTOOLS).

## Supplementary information

Supplementary Information

## Data Availability

All data used in this analysis are available as follows. FBCO and FC_deep_ data are available through the Faroe Marine Research Institute, see http://www.envofar.fo/index.php?page=climate. ERA-interim data are available through ECMWF, see https://www.ecmwf.int/en/forecasts/datasets/reanalysis-datasets/era-interim. Satellite altimetry data are now available through E.U. Copernicus Marine Service Information, see http://marine.copernicus.eu/services-portfolio/access-to-products/. NEMO data is available on CEDA server, see http://opendap4gws.jasmin.ac.uk/thredds/nemo/root/nemo_catalog.html. SB, SY and SC data are available upon request from the Faroe Marine Research Institute (karinl@hav.fo), while SG data is available upon request from Marine Scotland Science (b.berx@marlab.ac.uk). MS Norröna data are available at http://po.msrc.sunysb.edu/Norrona/.

## Code availability

The Lagrangian code can be accessed from GitHub, see https://github.com/TRACMASS/tracmass. Computer codes used to analyse the data are available from the corresponding author on reasonable request.
