# Supplementary material for: Discovery of an unrecognized pathway carrying overflow waters toward the Faroe Bank Channel
Source: Nat Commun. 2020 Jul 24;11:3721. doi: 10.1038/s41467-020-17426-8 (PMC7382466; doi:10.1038/s41467-020-17426-8)
Supplement: Supplementary file 1 — Supplementary Information [file 41467_2020_17426_MOESM1_ESM.pdf]

SUPPLEMENTARY INFORMATION

DISCOVERY OF AN UNRECOGNIZED PATHWAY CARRYING OVERFLOW WATERS TOWARD THE FAROE BANK CHANNEL

Léon Chafik[1]*, Hjálmar Hátún[2], Joakim Kjellsson[3,4], Karin Margretha H. Larsen[2], Thomas Rossby[5], Barbara Berx[6]

** corresponding author: leon.chafik@misu.su.se*

1 Department of Meteorology and Bolin Centre for Climate Research, Stockholm University, Stockholm, Sweden

2 Faroe Marine Research Institute, Tórshavn, Faroe Islands.

3 GEOMAR Helmholtz Zentrum für Ozeanforschung Kiel, Kiel, Germany

4 Christian-Albrechts-Universität, Kiel, Germany

5 Graduate School of Oceanography, University of Rhode Island, Kingston, USA

6 Marine Scotland Science, Aberdeen, Scotland, UK

Supplementary Figures

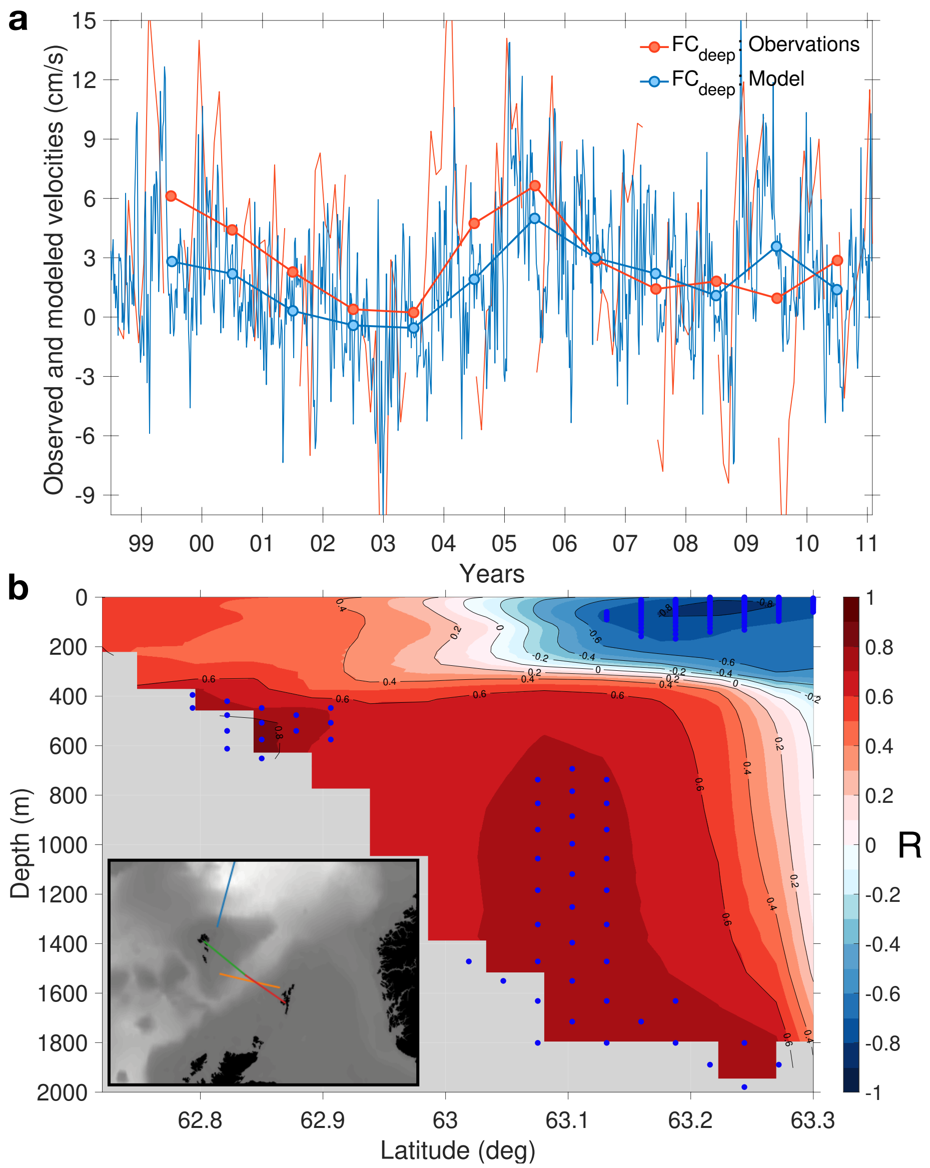

Supplementary Figure 1. (a) Comparison between current-meter measurements (zonal component) of the FC_{deep} (red) and zonal velocities from the model (blue) at the same location. The thin green line is the FC_{deep} monthly time series and the blue line is the 5-day zonal velocities from the model. The thick green and blue lines are the annual mean FC_{deep} and model velocities. The associated correlation is larger than 0.8 ($p < 0.05$, see panel b). (b) Correlation between the annual mean FC_{deep} and the simulated velocities (1999-2010; Methods) at a section extending from the Faroes and into the Norwegian Sea (see blue section in the inset). The inset also shows the FSC section (orange) from where the modeled current velocities (Fig. 5) and water mass properties (Fig. 7) are extracted, and the eastern (red) and western (green) sections used for the model transport calculations (Fig. 6). Stippling indicate 95% significance.

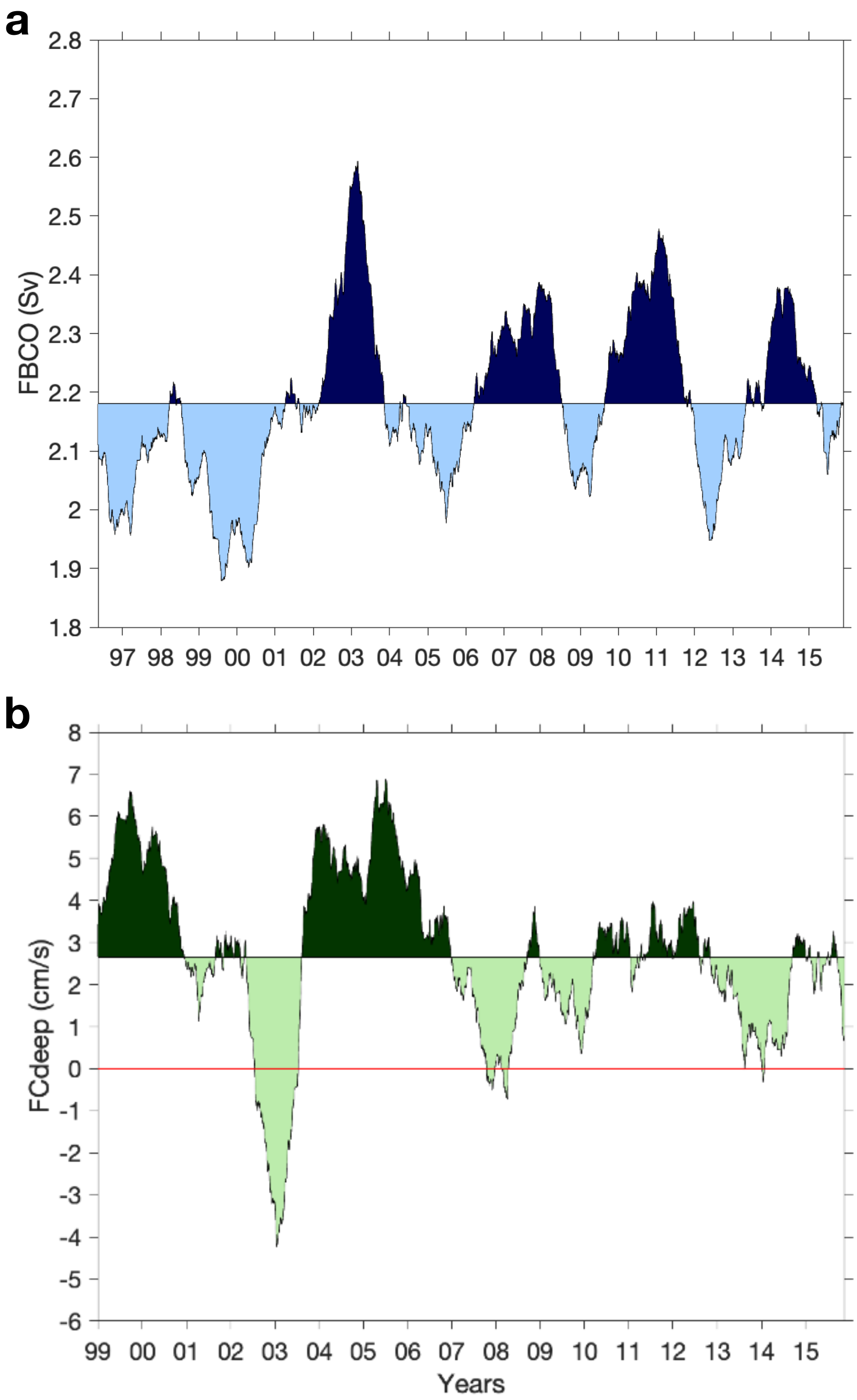

Supplementary Figure 2. (a) The overflow volume transport through the Faroe Bank Channel for the period 1996-2016 (shading) with the mean as a baseline (dark blue = above the mean, light below = below the mean). The time series has been smoothed with a 360-day running mean. Note that the FBCO was anomalously strong during 2002-2003. (b) The actual velocities from the FC_{deep} for the 1999-2016 period (shading) with the mean as a baseline (dark green = above the mean, light green = below the mean). The time series has been smoothed with a 360-day running mean. Note that the FC_{deep} reversed direction during 2002-2003.

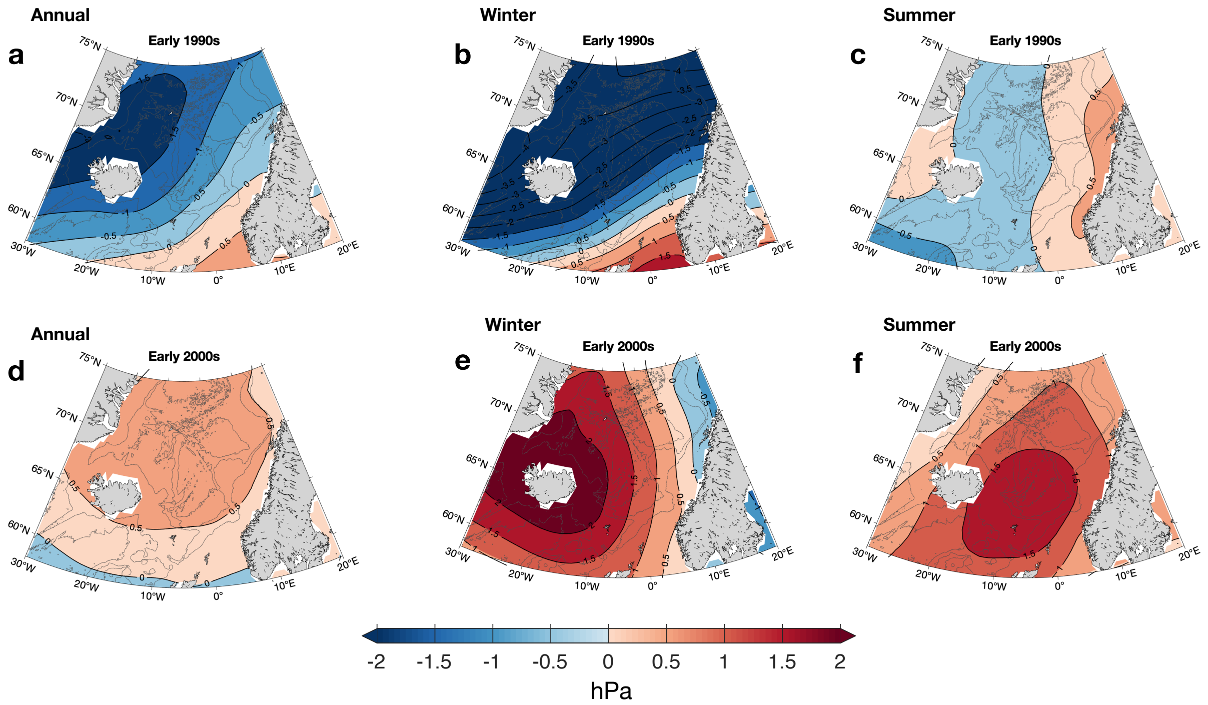

Supplementary Figure 3. Sea-level pressure anomalies (hPa) for the two periods, (a-c) 1990-1994 and (d-f) 2000-2004. (a/d) annual-, (b/e) winter- and (c/f) summer-mean sea-level pressure anomaly. The monthly sea-level pressure fields have been deseasonalized and detrended before the analysis. The contrasting sea-level pressure patterns reflect a generally cyclonic atmospheric circulation regime during the early 1990s and an anti-cyclonic regime during early 2000s, respectively. These atmospheric circulation regimes are found to excite the different pathways feeding the FBCO (see Fig. 4).

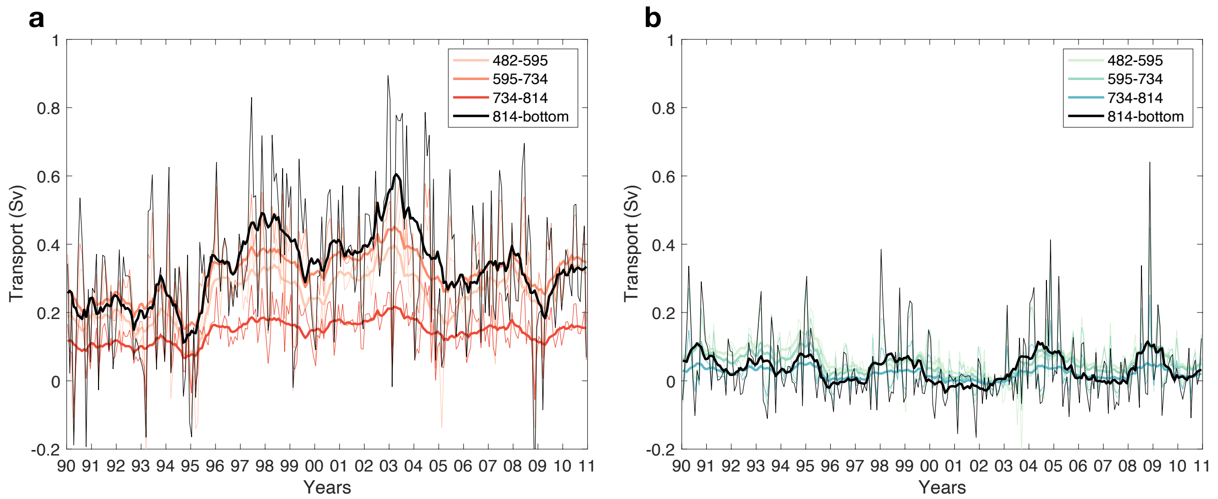

Supplementary Figure 4. The modeled southwestward directed volume transport calculated for four model layers (m, see labels) in the (a) eastern boundary and (b) western boundary (red and green sections, respectively, Supplementary Figure 1, inset) in the FSC (Methods). The largest transports are carried by the near-bottom flow (814 m – bottom, black curve in the left panel) and at middepth (light brown lines), both which are associated with the FSCJ (see Fig. 5).

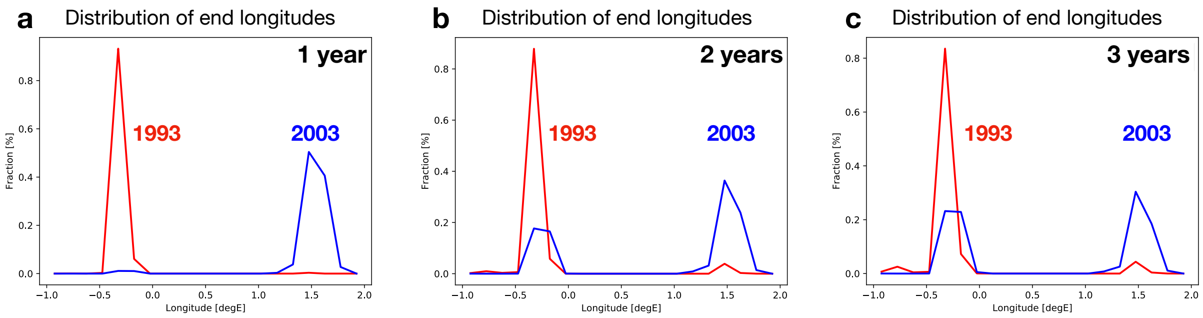

Supplementary Figure 5. Sensitivity analysis of particle arrival time (panel a = 1 year, panel b = 2 years, panel c = 3 years) to the northern boundary (cf. Fig. 4) where they are counted. The analysis demonstrates that water feeding the FBCO approaches the Faroe-Shetland Channel along two pathways: One western path north of the Faroes and one eastern path from the Norwegian slope. The two different starting years (1993 in red and 2003 in blue) further highlight the important role played by atmospheric forcing in modulating the pathways.
